# Response of *Corchorus olitorius* Leafy Vegetable to Cadmium in the Soil

**DOI:** 10.3390/plants9091200

**Published:** 2020-09-14

**Authors:** Sibongokuhle Ndlovu, Rajasekhar V.S.R. Pullabhotla, Nontuthuko R. Ntuli

**Affiliations:** 1 Department of Botany, University of Zululand (Main Campus), Private Bag X1001, KwaDlangezwa 3886, South Africa; sbongokuhlendlovu71@gmail.com; 2 Department of Chemistry, University of Zululand (Main Campus), Private Bag X1001, KwaDlangezwa 3886, South Africa; PullabhotlaV@unizulu.ac.za

**Keywords:** cadmium toxicity, *Corchorus olitorius*, hormesis, phytoremediation, morphological traits

## Abstract

*Corchorus olitorius*, a leafy vegetable with high nutrient content, is normally collected from the wild, in areas that are prone to cadmium (Cd) toxicity. However, studies on how Cd accumulation affects vegetative and reproductive traits of leafy vegetables in South Africa are limited. Therefore, this study tested the effect of Cd accumulation on *C. olitorius* morphological traits. Plants were grown under various Cd concentrations and studied for variation in vegetative and reproductive traits as well as accumulation in roots and shoots. Plants exposed to 5 mg/kg Cd had longer roots with higher moisture content, heavier fresh and dried stems, as well as dried leaves, which indicated a hormetic effect in *C. olitorius* after exposure to low Cd concentration in the soil. Again, plants treated with 5–10 mg/kg Cd, accumulated toxic (>10 mg/kg dry weight) Cd within shoots and roots, with minor morphological alterations. Plants could survive, with some morphological defects, Cd toxicity up to 20 mg/kg in soil. Only plants exposed to 5 mg/kg could reproduce. Cd accumulation increased with an increase in the soil, with higher accumulation in shoots. The translocation factor was high (>1) in all Cd concentrations. In conclusion, *C. olitorius* can accumulate toxic Cd, and yet grow and reproduce either normally or better than the control. The proposed dose of Cd that induces hormesis in *C. olitorius* is 5 mg/kg in the soil. Therefore, *C. olitorius* is suitable for phytoremediation of Cd contaminated soils, but unsafe for consumption when it grows in such areas.

## 1. Introduction

*Corchorus olitorius* L., commonly known as Jute mallow, is an annual erect herb that belongs to the Malvaceae family [1], which grows on roadsides [2], fields, and home gardens [3]. Its leaves and tender stems are rich in vitamin A and C, beta carotene, folic acid, iron, calcium, and several phenolic antioxidative compounds [1]. Cooked *C. olitorius* vegetables are also recommended for pregnant and nursing mothers because of their high iron content [2]. *Corchorus olitorius* leaves possess antidiabetic, antioxidant, and hepatoprotective properties, and thus they are used for different ailments in folk medicine [4]. The plant is also harvested for its fiber [5].

Cadmium (Cd) is a divalent cation and a metallic trace element that is non-essential to living organisms [6,7], but can be easily absorbed by roots and transported to the aboveground parts of plants [8]. Cd is a non-biodegradable and persistent heavy metal that can either occur naturally through volcanic eruption or deposited into the soil as a result of many anthropogenic activities [6]. According to the World Health Organization (WHO) and the European Union (EU), the maximum permissible limit of Cd in agricultural soil is 3.0 mg/kg [9]. Cadmium is one of the most widespread heavy metals that can accumulate in the food chain and become highly toxic to humans and animals consuming plants from contaminated areas [10], as well as plants growing in such areas [6]. Cadmium toxicity disorders in humans include cardiovascular, nervous, placenta, and pancreas physiological damages [7].

Hormesis or hormetic effect is a bi-phasic concentration-response phenomenon that is characterized by a stimulatory effect of low doses and inhibitory effect of high doses of toxic substances, such as heavy metals [6,8,11]. This phenomenon is represented as a U- or J-shaped or inverted U- or J-shaped dose-response curves depending on the variable under evaluation [6,11,12]. The stimulatory effect which results in cell proliferation, growth, and longevity, is presented as an inverted U-shaped graph [12]. Hormesis in some plants exposed to low doses of toxic substances protects them against stress and increases their growth, yield, and productivity [13,14,15]. Exposure of plants to Cd normally interferes with their morphology and physiology, and thus triggers negative effects in their growth and productivity [11], where reproductive traits are more sensitive than vegetative traits [16,17]. However, when some plants are exposed to Cd, they show an improved root and shoot performance, including photosynthetic components, despite accumulating Cd that is toxic towards animal and human consumption in their roots and shoots [6,11,18].

Cadmium is absorbed from the soil by the roots and is transported to the stem and leaves in the apoplastic and symplastic pathways [16,19]. A large amount of accumulated Cd is normally stored in the roots and little is transported to the aerial parts of the plants [16,20]. However, this varies among plants as described by a translocation factor, which is described as the concentration of metals in the shoots of the plants in relation to the roots [19]. The factors that affect Cd uptake from the soil include the level of Cd concentration and its bioavailability in the soil [19]. The increase in concentration in the soil results in an increase in the Cd content in the roots as well as its translocation to the shoots [16]. The bioavailability is moderated by the soil pH, organic matter content, soil temperature, and concentration of other elements in the soil [16].

Vegetables have different responses to Cd stress, even among cultivars and varieties of the same species [21]. Leafy vegetables have a potential to accumulate higher amounts of Cd in their edible parts when compared to tubers and root vegetables, and thus they easily intoxicate humans that consume them [21,22]. Plants, including vegetables, with an ability to grow and yield successfully after they have accumulated high amounts of toxic heavy metals, have the potential for phytoremediation purposes [23]. Phytoremediation is a process of using plants to remove or stabilize heavy metals from the soil in order to reduce their toxicity [24].

*Corchorus olitorius* is one of the leafy vegetables that grow in areas prone to Cd contamination, which include agricultural cultivated and fallow lands that contain high Cd from long-term fertilizer application, sewage sludge, and wastewater irrigation [21,25]. However, studies on *C. olitorius’* potential accumulation and response towards toxic Cd amounts in the soil are limited in the country and elsewhere. Such a study is essential to indicate morphological, physiological, growth, and yield responses of *C. olitorius* plants that grow in Cd-contaminated areas and have accumulated toxic Cd concentrations within their edible plant parts in comparison to plants from uncontaminated areas. Results from such a study also determine the possibility of this species for phytoremediation purposes and also caution the consumers about harvesting *C. olitorius* vegetable from Cd contaminated areas. Therefore, the objective of this study was to determine the effect of Cd concentration in the soil on vegetative and reproductive traits of *C. olitorius* and on its Cd accumulation potential. It is hypothesized that *C. olitorius* exposed to low but toxic Cd concentrations in the soil can accumulate toxic amounts within its edible plant parts but grow and yield better than or similarly as uncontaminated plants.

## 2. Results

### 2.1. Variation in Vegetative and Reproductive Traits of Corchorus olitorius after Cadmium Treatment

Vegetative traits of *C. olitorius* responded differently to the application of Cd to the soil at varying concentrations (Table 1). Significant (*p* < 0.05) differences were recorded within each trait when compared among Cd treatments and/or with untreated plants. The highest (26.9) and lowest (1.6) coefficient of variation were recorded in the number of branches and stem moisture content, respectively. Germination percentage and stem moisture content of plants exposed to Cd treatments differed from each other but were all relatively similar to the control. Plants from soil treated with 5 mg/kg germinated better than those exposed to 20 mg/kg, whereas stems of plants treated with 10 mg/kg had better moisture content than those of plants grown under 5 mg/kg Cd concentration.

Exposure of plants to 5 mg/kg Cd promoted the formation of longer roots, heavier fresh and dried stems, and heavier dried leaves compared with the control, but higher concentrations had an opposite effect on these traits. Again, plants exposed to 5–10 mg/kg Cd had higher root moisture content than the control. On the contrary, the same treatment (5 mg/kg Cd) resulted in shorter plants with thinner stems, fewer leaves that were smaller, and lower chlorophyll content than untreated plants, where the effect increased as the Cd concentration increases.

Cadmium application of 10 mg/kg was associated with lighter dried roots when compared with the control and plants exposed to 5 mg/kg Cd. Again, shorter roots, and lighter fresh leaves and roots corresponded with 15 mg/kg Cd treatment in relation to control and 5–10 mg/kg Cd-treated plants. The plants treated with 20 mg/kg Cd had fewer branches and leaves with lower moisture content than untreated plants. A significant reduction in seed germination was recorded only for plants exposed to 20 mg/kg Cd when compared with 5 mg/kg Cd.

Only plants treated with 5 mg/kg Cd produced reproductive traits (Table 2). Cadmium-treated plants had shorter pods with a heavier total and 100-seed mass than untreated plants. However, treated and untreated plants produced similar numbers of pods per plant, pod mass, and numbers of seeds per pod.

### 2.2. Cadmium Accumulation and Translocation

*Corchorus olitorius* accumulated Cd differently within its dried roots and shoots in response to different Cd concentrations in the soil, when measured at both seedling and maturity stages (44 and 85 days after sowing, respectively) (Table 3). The coefficient of variation was higher in roots (11.8) than in shoots (3.8). Cadmium was not detected in control plants. Exposure of the plants to 20 mg/kg Cd resulted in the most Cd accumulation in the shoots (1942.5 mg/kg) at maturity, but the least in the roots (122.5 mg/kg) at an immature stage, when compared with all plant parts at different Cd concentrations. Generally, the accumulation of Cd within the plant increased as it increases in the soil. However, immature roots had lesser Cd accumulations on plants exposed to 20 mg/kg Cd application.

The exposure of *C. olitorius* to different Cd concentrations in the soil resulted in higher Cd accumulation in shoots than in roots (Table 3). However, plants treated with 10 mg/kg Cd had a similar distribution of Cd between roots and shoots at an immature stage of growth. Accumulation of Cd also differed within roots and shoots when they were each compared at immature and mature stages of growth. Exposure of plants to 5 mg/kg resulted in a similar amount of Cd in roots at both immature and mature stages of growth, whereas plants treated with 10 mg/kg accumulated more Cd in their roots at seedling stage than at maturity. Further, the treatment of plants with 15 and 20 mg/kg Cd was associated with lesser accumulation of Cd in roots at the seedling stage than at maturity. In shoots, only plants exposed to 5 mg/kg Cd had similar Cd content at both seedling and maturity stages but shoots from plants exposed to other concentrations accumulated lesser Cd at immaturity than at maturity.

In all treatments, the cadmium translocation factor (Cd root to shoot ratio) was greater than one, which indicated that shoots accumulated more total Cd than roots (Table 4). In immature plants, the translocation factor increased with an increase in Cd content in the soil, but the opposite was recorded in mature plants. Seedlings exposed to 20 mg/kg Cd had the highest translocation factor (11.66), whereas those treated with 10 mg/kg had the lowest (1.16), but the latter was significant when compared with mature plants exposed to 5–10 Cd treatment.

Translocation of cadmium also differed between immature and mature plants that were exposed to the same treatments. Plants treated with 5 and 10 mg/kg Cd had higher translocation factors for mature plants than seedlings. However, immature plants exposed to 20 mg/kg had better root-shoot Cd translocation than mature plants. Further, similar root-shoot Cd translocation was recorded at both stages of growth for plants exposed to 15 mg/kg Cd.

## 3. Discussion

### 3.1. Effect of Cadmium on Morphometric Features of Corchorus olitorius

The insignificant changes in the germination rate and stem moisture content of *Corchorus olitorius* seeds sown in toxic (5–20 mg/kg) Cd-treated soils compared with the control indicate that this plant can germinate successfully and retain its sappiness even in Cd concentrations far above the maximum allowable limits of 3 mg/kg in soil [26]. A comparison of seed germination rates among treated soils also reveals that *C. olitorius* can tolerate toxic Cd concentrations until the maximum of 15 mg/kg. This tolerance might be related to the impermeability of seed coats to this heavy metal after imbibition and which allows them to possibly avoid the over-accumulation of Cd in the germinating seeds [27]. However, a significant decline in seed germination percentage was recorded in *Phaseolus vulgaris* seeds treated with 5 mM Cd [27], and in *Ocimum basilicum* seeds exposed to 2 mg/L Cd [28]. This explains differences in Cd tolerance potential among different species, where some of the herbaceous plants have high survival potential and resilience to environmental stress, and can thus pioneer and survive contaminated areas [29]. This *C. olitorius* tolerance towards Cd also shows its phytoremediation potential for Cd contaminated areas, as some plants can germinate vigorously in areas with high Cd concentrations, while others fail [17]. A reduction in the germination of *C. olitorius* seeds exposed to Cd concentrations above 20 mg/kg is probably related to the impairment of water uptake, which limits its availability to the embryo and therefore inhibits seed germination [17].

The formation of longer roots, heavier fresh and dried stems, and heavier dried leaves in plants exposed to 5 mg/kg as well as roots with higher moisture content on 5–10 mg/kg Cd-treated plants can be attributed to the fast growth rate and high proliferation potential of *C. olitorius* as an herbaceous plant in contaminated areas [29]. Additionally, the production of leaves with heavy dry mass might indicate that *C. olitorius* formed leaves with larger mesophyll cells when they were exposed to Cd [17]. The stimulation of root growth and moisture content as well as stem and leaf biomass when *C. olitorius* was exposed to low concentrations of Cd represents the hormetic response of this species towards Cd availability in the soil [11,14,15]. Similarly, the roots of *Lonicera japonica* elongated when the plant was exposed to 5–25 mg/kg Cd in the soil when compared with the control and were then retarded by higher concentration [30]. Elongation of *C. olitorius* roots in the presence of 5 mg/kg Cd was probably achieved by the quick activation of defensive mechanisms, which was necessary to maintain viability and function of cells while they adapt to the new Cd-rich environment [11]. Therefore, the production of shorter roots in *C. olitorius* plants treated with 15 mg/kg Cd probably indicates the limit of Cd tolerance for this species. Application of 100 µM Cd also retarded the length of *Miscanthus sacchariflorus* roots [31]. In *Paspalum fasciculatum* grown in 15–50 mg/kg Cd contaminated soil, root biomass increased at 30 mg/kg, whereas stem and leaf biomass of all treated plants was similar to the control, at 30 days after sowing [32]. Further, as a hormetic response towards toxic Cd, root, stem, and leaf dry weights of *L. japonica* were increased by the application of 2.5, 2.5–5, and 2.5–25 Cd, respectively, in relation to the control [30].

The reduction in root length, leaf and root fresh mass at 15 mg/kg, and in the number of branches and leaf moisture content at 20 mg/kg Cd applications, probably means that these traits are not easily affected by toxic Cd content in the soil. This shows a tolerable concentration range of 5–15 and 5–20 mg/kg for non-essential toxic Cd element in *C. olitorius*, where beyond its growth was inhibited, thus displaying both hormetic and toxic responses towards Cd contamination in the soil [18]. These findings are related to insignificant changes in the number of new shoots in *Typha domingensis* plants exposed to toxic Cd in the soil [33]. Similarly, the root length of *Hylotelephium spectabile,* a Cd-accumulating species, was not affected by exposure to 5–10 mg/L Cd [34]. A decline in root and shoot fresh mass as a result of increasing Cd contents in the soil was also reported in *Lactuca sativa* [35]. Reduction in root length can be attributed to high Cd levels that might have led to inhibition of mitosis in cells of root tips, which decreased the production of root hairs, and the formation of asymmetrical epidermal and cortical cells as well as intercellular air spaces [34].

Although plant height, stem girth, number of leaves, leaf area, and leaf chlorophyll content were retarded by 5 mg/kg Cd treatment, these traits remained relatively the same in higher treatments (10–20 mg/kg Cd). Their tolerance towards high Cd concentrations is indicative of species resistance towards toxic Cd concentrations, and thus a phytoremediation property of this plant [23]. Generally, improvement in leaf chlorophyll content is related to photosynthate production and consequent biomass accumulation [11]. However, exposure of *C. olitorius* to 5 mg/kg Cd reduced the leaf chlorophyll content, but either enhanced or did not affect leaf and stem biomass. Chlorosis in *C. olitorius* leaves at 5 mg/kg probably resulted from reduced photosynthetic pigments that caused the destruction of chloroplast structures in response to Cd toxicity [36]. However, the resistance at higher Cd concentrations was possibly caused by the storage of more Cd in the epidermal cells than in mesophyll cells of the leaves, and thus avoid damaging of photosynthetic apparatus in the leaf [37]. Although chlorosis was evident in exposure to lower Cd concentration (5 mg/kg), the possible normal photosynthesis process in the mesophyll cells because of Cd sequestration in vacuoles and cell walls of the leaves [21] might have resulted in high leaf and stem biomass of *C. olitorius*. Similar to *C. olitorius*, 5 mg/kg Cd resulted in shorter plants, fewer leaves, and lower leaf chlorophyll content in *Lactuca sativa*, when compared with the control [38]. Additionally, *Eruca sativa* plants grown in 100–200 mg/kg Cd formed thinner stems and fewer, small-sized leaves with lower chlorophyll content than the control [39]. However, 10 and 50 µM Cd applications did not affect the number of leaves, leaf size, and leaf chlorophyll content in *Typha domingensis* [33].

*C. olitorius* stems with higher moisture content when exposed to 10 mg/kg Cd than to 5 and 15 mg/kg Cd showed a hormetic curve, where moisture content was stimulated by low concentrations of non-essential, toxic elements [18]. The manifestation of hormesis in stem moisture content followed the priming, where exposure to a low dose of 5 mg/kg Cd preconditioned *C. olitorius* stems for tolerance and higher performance in higher (10 mg/kg) Cd dose [14]. The formation of reproductive traits only in plants exposed to 5 mg/kg Cd probably indicates that this plant has low Cd toxic tolerance for reproduction purposes, thus this phase is highly susceptible [17]. This scenario confirms that reproductive traits are more sensitive to Cd toxicity than vegetative traits [16,17]. However, the normal growth of vegetative features can make *C. olitorius* toxic for consumption when harvested from soils with higher concentrations [25]. Again, this concentration is far above the maximum allowable content in the soil (3 mg Cd per kg of soil) [9], which means that this plant can grow and reproduce successfully under Cd concentrations of ≥2 times the maximum allowable soil concentration.

### 3.2. Phytoremediation Potential of C. olitorius

In all soil concentrations (5–20 mg/kg), Cd was present in the roots and shoots, clearly showing the ability of *C. olitorius* leafy vegetable to translocate and accumulate Cd in all plant parts. Therefore, intercropping *C. olitorius* with other Cd-sensitive crops in Cd-contaminated soils can possibly assist them to improve their growth and yield due to the increase in soil exchangeable Cd with intercropping [21]. *C. olitorius* is normally declared as a weed because of its wild-state occurrence [2,3], thus its intercropping with sensitive crops in Cd contaminated areas can be beneficial because weeds normally outperform the cultivated plants [15], and it can efficiently extract Cd from the soil. However, the same outperformance ability of this plant can pose a threat in co-existing with other crops in a given field condition, where *C. olitorius* may outcompete these crops in a dose-response spectrum [15]. This plant accumulated a range from 812.5–1942.5 mg/kg of Cd in shoots, at all stages of growth when grown in 5–20 mg/kg Cd, which is far above the permissible 0.2 mg/kg dry weight for human consumption [9].

The translocation factors of greater than one at exposure to all Cd concentrations in the soil also indicate higher accumulation of Cd in shoots than in roots, which makes *C. olitorius* desirable for phytoremediation applications but should not be consumed when collected from Cd contaminated areas [25]. In its use for phytoremediation purposes, *C. olitorius* can be recommended for phytoextraction because it can grow well in Cd contaminated soil, remove it, and accumulate it within its plant parts [24,40,41]. Hormetic response of *C. olitorius* to Cd treatment as represented by the formation of longer roots, heavier fresh and dried stems, as well as dried leaves at 5 mg/kg Cd treatment, and higher moisture content in stems and roots of plants exposed to 5 and 10 mg/kg Cd, after accumulating toxic Cd content, was probably a result of compartmentation of toxic Cd away from essential metabolic activities [25]. Comparison of Cd accumulation in roots at different stages of growth showed that plants exposed to 15 and 20 mg/kg Cd in the soil accumulated more Cd at maturity than at the immaturity stage. This could have resulted from the redistribution of Cd from shoots to roots through phloem as part of the detoxification process [19].

In *C. olitorius*, the translocation factors from roots to shoots at immaturity increased with an increase in Cd concentration in the soil. Although, immature plants have accumulated higher Cd in their shoots they showed either stimulated and/or similar growth as untreated plants, particularly at exposure to 5 mg/kg Cd in the soil, which fits well with the hormesis concept [6]. To cope with Cd stress, *C. olitorius* might have undergone the sequestration of Cd in leaf vacuoles, which are metabolically inactive [21]. A similar increase in translocation factors from roots to shoots was obtained in *Salix mucronata* exposed to 20–60 mg/kg Cd [42].

Lower translocation of Cd from roots to shoots in mature than immature *C. olitorius* plants exposed to 20 mg/kg Cd, was correlated with high Cd retention in the roots than shoots of this plant. This retention was probably a pronounced compartmentalization of Cd in the cell walls or vacuole of the roots in order to tolerate Cd toxicity, and thus sequester it from the root cytoplasm and prevent its translocation to the shoots [16,25]. However, an increase in roots to shoots translocation factor at 5 and 10 mg/kg Cd-treatment at maturity than immaturity stage probably signifies that in *C. olitorius*, Cd is primarily compartmentalized in cell walls and is translocated and stored in all plant parts, regardless of the growth stage, similar to *Impatiens glandulifera* [37].

## 4. Materials and Methods

### 4.1. Soil Properties, Seed Sourcing, Study Area and Experimental Design

A black humus-rich soil, where *Corchorus olitorius* normally grows, was collected from the university’s farm at 30–60 cm soil depth and had its properties analyzed using the method described by Manson and Roberts [43] (Table 5). Soil samples were air-dried at room temperature; where they were spread out in drying trays and air was forced over them. When dry, the samples were crushed between rubber belts on a soil crusher and passed through a 1-mm sieve. Material coarser than 1 mm that cannot be crushed (such as stones, gravel, and concretions), were discarded. Soil samples were scooped into trays which each contained 11 PVC cups (capacity 70 mL); a tray was used for nine unknown samples, one standard soil sample (for quality control), and one blank. For operations such as dispensing and stirring and for quality control, batches of three trays (27 samples, three unknowns, and three blanks) were used. Multiple dispensers and diluter/dispensers were used to dispense aliquots of extractant or reagent to three samples at a time.

Soil samples were analyzed on a volume rather than a mass basis. To enable the conversion of the results to a mass basis, the mass of a 10-mL scoop of a dried and milled sample was measured and the calculated sample density was reported. For the determination of pH, 10 mL of soil was scooped into sample cups. Then, 25 mL of 1 M KCl solution was added and the suspension was stirred at 400 revolutions per minute (r.p.m.) for 5 min using a multiple stirrer. The suspension was allowed to stand for about 30 min, and the pH was measured using a gel-filled combination glass electrode while stirring. De-ionised water was substituted for the 1 M KCl solution if pH (water) was required.

*Extractable (1 M KCl) calcium and magnesium*: A 2.5 mL of soil was scooped into sample cups. Then, 25 mL of 1 M KCl solution was added and the suspension was stirred at 400 r.p.m. for 10 min using a multiple stirrer. The extracts were filtered using Whatman No.1 paper. A 5 mL of the filtrate was diluted with 20 mL of 0.0356 M SrCl2, and Ca and Mg determined by atomic absorption. To determine extractable acidity, 10 mL of the filtrate is diluted with 10 mL of de-ionised water containing 2–4 drops of phenolphthalein, and titrated with 0.005 M NaOH.

*Extractable (Ambic-2) phosphorus, potassium, zinc, copper and manganese*: The Ambic-2 extracting solution consisted of 0.25 M NH4CO3 + 0.01 M Na2EDTA + 0.01 M NH4F + 0.05 g L^−1^ Superfloc (N100), adjusted to pH 8 with a concentrated ammonia solution. A 25 mL of this solution was added to 2.5 mL soil, and the suspension was stirred at 400 r.p.m. for 10 min using a multiple stirrer. The extracts were filtered using Whatman No.1 paper. Phosphorus was determined on a 2 mL aliquot of filtrate using a modification of the Murphy and Riley [44] molybdenum blue procedure [45]. Potassium was determined by atomic absorption on a 5 mL aliquot of the filtrate after dilution with 20 mL de-ionised water. Zinc, Cu, and Mn were determined by atomic absorption on the remaining undiluted filtrate.

*Estimation of clay content by near-infrared spectroscopy and of organic matter by the Walkley–Black method*: Clay content was estimated for all soil samples routinely analyzed using a combination of near-infrared reflectance using the air-dry, milled soil samples, and the measured sample density. Organic matter was based on the Walkley–Black procedure [46], which measured the readily oxidizable organic carbon. The organic matter was oxidized by potassium dichromate in a sulphuric acid medium. The excess dichromate was determined by titration with standard ferrous sulphate solution.

Seeds of *Corchorus olitorius L.* were sourced from the Agricultural Research Council in Roodeplaat, Pretoria. Subsequent experiments were conducted at the University of Zululand (28.85416° S, 31.84565° E), Department of Botany, in a rain-free environment. Twenty-liter plastic pots were filled with soil mixed with Cd in a form of a substrate Cadmium nitrate tetrahydrate [Cd (NO_3_)_2_4H_2_O] (Sigma-Aldrich, Merck, South Africa) at a rate of 0, 5, 10, 15, and 20 mg/kg of soil as modified from Khodaverdiloo et al. [47]. Pots were irrigated with deionized water to field capacity, covered with plastic bags, and were incubated (aged) for ten days. All the tested Cd values were above the threshold value of Cd in agricultural soils (>3 mg/kg) [9]. The experiment was laid out in a randomized complete block design of five pots per treatment, where each pot served as a replicate. Ten seeds of *C. olitorius* were germinated in each pot and later thinned into one plant per pot, followed by application of 2:3:4 (27) NPK fertilizer (Kynoch Fertilizer, South Africa) at a rate of 1 g/kg of soil. Plants were regularly irrigated with deionized water (50 mL per pot), which was then collected from the base of the pots and reused to irrigate, to avoid nutrient or heavy metal loss. Each pot had a separate catch pan to recapture the effluent in order to avoid possible cross-contaminations among replicates.

### 4.2. Measurement of Agronomic Traits

Germination percentage was recorded at seven days after sowing (DAS), before thinning. All vegetative traits were measured at 44 DAS, whereas the number of branches and all reproductive traits were measured at 85 DAS, in quintuplicate. Plant height (cm) was measured from the soil level to the tip of the stem using a ruler. The numbers of leaves and branches were counted manually. Vernier calipers were used to measure stem width (mm) at 10 cm from the soil level. Leaf area (length × width) (cm^2^) was recorded on the fourth leaf from the apex using a ruler. The leaf chlorophyll content of the fifth oldest leaf (from the apex of the main stem) was captured with a chlorophyll content meter (CCM-200 *plus*, Opti-Sciences, ADC BioScientific Ltd., Hoddesdon, UK). Five different spots were randomly measured in the leaf and provided the average leaf chlorophyll content. Each spot measured by CCM-200 were 1 cm diameter circles, which calculated the optical absorbance in two different wavelengths, namely, 653 nm (chlorophyll) and 931 nm (near infra-red), and provided the chlorophyll content index (CCI) value.

Plants were uprooted, washed with distilled water and blot-dried with a paper towel, and had their root length (cm) measured from the root tip to the base of the stem using a ruler. The fresh mass (g) of roots, stems and leaves of uprooted plants were measured separately. Separated plant parts were oven dried at 60 °C until constant dry mass. The dry mass (g) and moisture content (%) of roots, stems and leaves were also determined separately. Moisture content was determined using the following formula: Moisture content (%) = [fresh mass – dry mass/fresh mass] × 100. The number of pods per plant was counted manually, and pod length (cm) and width (cm) determined with Vernier calipers. Seed traits were determined from five pods per plant per treatment. The number of seeds per pod was counted manually; total seed mass (g) per pod and 100-seed mass (g) in each pod were also recorded.

### 4.3. Cadmium Content of Plant Parts

Plants were analyzed for cadmium accumulation in roots and shoots (stems and leaves) at both seedling (44 DAS) and termination or maturity (85 DAS) stages. Three instead of five pots were selected for harvesting and each pot was used as a replicate for each plant part. This was modified because the procedure for determining Cd content in plants was destructive. Plants were carefully uprooted, thoroughly washed with distilled water, and blot-dried with a paper towel. Plants were then separated roots and shoots. Each plant part (in three separate replicates) was cut into pieces and further dried in an oven at 80 °C until they reached a constant weight. Dried samples were ground into powder and packed in air-tight plastic containers and stored in a fridge (−4 °C) for further analysis. Cd content was determined according to the method determined by Ganje and Page [48] with some modifications. One gram of each milled sample was dissolved in 5 mL of 60% hydrochloric acid and 10 mL of 70% nitric acid, and then digested at a moderate temperature of 50 °C until white fumes evolved, and the solution changed to a brownish colour. The heat was further intensified for a few minutes to expel most of the HCl. Then 50 mL distilled water was added, heated for a few minutes, and allowed to cool. The solution was filtered through a Whatman’s No. 1 paper into a transparent plastic container and was allowed to settle for a few minutes, for Cd to be aspirated accordingly. The digested sample was analyzed for cadmium concentration using an atomic absorption spectrometer (AAS) (Perkin Elmer AAnalyst 100). To find Cd concentration, all AAS readings were multiplied by 1000 to convert the values from grams to kilograms: [Cd] in mg/kg = ASS reading × 1000.

The translocation factor (TF) was calculated as the ratio of the total Cd content in the shoots to the total content in the roots. $\mathrm{TF}=\frac{\mathrm{Cd}\;\mathrm{shoots}}{\mathrm{Cd}\;\mathrm{roots}}$.

### 4.4. Statistical Analysis

Data were subject to analysis of variance (one-way ANOVA) in GenStat 12.1 version (VSN International, England, UK). Means were separated using Tukey’s multiple range tyest in GenStat at a 5% level of significance. Correlation matrix analysis also determined the relationship between vegetative traits.

## 5. Conclusions

*Corchorus olitorius* can grow successfully in toxic Cd concentrations of up to 15 mg/kg in the soil, but can only reproduce at the maximum 5 mg/kg Cd toxicity. Root length, stem fresh and dry mass, leaf dry mass, and root moisture content were stimulated by exposure to 5 mg/kg Cd, but were inhibited at high concentrations, confirming a hormetic response towards Cd toxicity. This species accumulated high amounts of Cd (122.5–1942.5 mg/kg Cd dry weight) within its roots and shoots at different stages of growth, where more Cd was translocated to the shoots, as represented by high translocation factors (>1), with less toxicity in vegetative traits. These features suggested that hormetic effects should be considered in phytoremediation of Cd contaminated soil and the dose of Cd in the soil that induces hormesis (stimulative effect) in *C. olitorius* is proposed as 5 mg/kg. Therefore, consumption of this vegetable species from areas prone to Cd contamination can be harmful to humans. Future research will focus on growth and yield variations in Cd-treated *C. olitorius* plants that are grown in different soil types under different climatic conditions.

## Figures and Tables

**Table 1:** Effect of cadmium in the vegetative traits of *Corchorus olitorius.*

Conc. (mg/kg)	GP (%)	RL (cm)	PH (cm)	SG (mm)	NB	NL	LA (cm^2^)	LCC (mg/cm^2^)	LFM (g)	SFM (g)	RFM (g)	LDM (g)	SDM (g)	RDM (g)	LMC (%)	SMC (%)	RMC (%)
0	95.0 ^a,b^	25.0 ^b^	44.4 ^a^	5.3 ^a^	10 ^a,b^	24.6 ^a^	45.7 ^a^	49.8 ^a^	7.8 ^a^	5.3 ^b^	1.3 ^a,b^	1.08 ^b^	0.7 ^b^	0.5 ^a^	84.9 ^a^	87.2 ^a,b,c^	58.6 ^d^
5	100.0 ^a^	31.8 ^a^	24.6 ^b^	4.4 ^b^	12 ^a^	15.4 ^b^	30.8 ^b^	20.4 ^b^	7.1 ^a^	6.8 ^a^	1.6 ^a^	1.6 ^a^	1.09 ^a^	0.4 ^a^	78.1 ^a,b^	84.7 ^c^	75.7 ^b^
10	93.0 ^a,b^	25.0 ^b^	16.0 ^c^	3.7 ^b,c^	8.2 ^b^	11.6 ^c^	14.7 ^c^	23.5 ^b^	7.5 ^a^	5.3 ^b^	1.2 ^b^	0.6 ^d^	0.56 ^c^	0.2 ^b^	86.1 ^a^	90.9 ^a^	84.4 ^a^
15	94.0 ^a,b^	21.2 ^c^	15.8 ^c^	3.6 ^c^	6.6 ^b,c^	12.4 ^b,c^	15.2 ^c^	17.0 ^c^	5.3 ^b^	4.7 ^b^	0.8 ^c^	0.9 ^b^	0.62 ^b c^	0.2 ^b^	83.2 ^a^	86 ^b,c^	66.2 ^c,d^
20	89.0 ^b^	12.2 ^d^	15.0 ^c^	3.2 ^c^	3.4 ^c^	11.6 ^c^	17.3 ^c^	10.4 ^d^	2.9^c^	2.0 ^c^	0.8 ^c^	0.85 ^c^	0.24 ^d^	0.18 ^b^	65.2 ^b^	89 ^a,b^	70.5 ^b,c^
Mean	94.2	19.2	23.2	4.1	8.0	15.1	24.7	24.2	6.1	4.8	1.2	1.0	0.6	0.31	79.5	87.6	71.1
CV%	5.7	5.5	10.4	10.3	26.9	12.6	13.2	6.9	4.3	5.8	9.2	6.7	5.3	11.7	8.0	1.6	3.9
LSD	6.85	1.38	2.95	0.5	2.5	2.4	3.8	2.2	0.5	0.5	0.2	0.13	0.06	0.07	11.98	2.6	5.2
*p*-value	<0.001	<0.001	<0.001	<0.001	<0.001	<0.001	<0.001	<0.001	<0.001	<0.001	<0.001	<0.001	<0.001	<0.001	0.02	0.004	<0.001

Different letters (a,b,c and d) indicate significant differences between cadmium concentrations for each trait (*p* < 0.05). Conc. (Cadmium concentration), GP (germination percentage), RL (root length), PH (plant height), SG (stem girth), NB (number of branches), LA (leaf area), NL (number of leaves), LCC (leaf chlorophyll content), LFM (leaf fresh mass), SFM (stem fresh mass), RFM (root fresh mass), LDM (leaf dry mass), SDM (stem dry mass), RDM (root dry mass), LMC (leaf moisture content), SMC (stem moisture content), and RMC (root moisture content).

**Table 2:** Variation in reproductive traits after treatment with cadmium.

Conc. (mg/kg)	PN	PL (cm)	PM (g)	SPP	TSM (mg)	100-SM (mg)
0	7.0 ^a^	6.3 ^a^	5.5 ^a^	118.5 ^a^	123.0 ^b^	104.0 ^b^
5	6.5 ^a^	6.2 ^b^	5.2 ^a^	115.8 ^a^	180.8 ^a^	160.0 ^a^
Mean	6.75	6.27	5.36	117.13	151.88	132.00
*p*-value	0.780	0.044	0.562	0.813	0.048	0.001

Different letters (a and b) indicate significant differences between cadmium concentrations for each trait (*p* < 0.05). Conc. (Cadmium concentration), PN (pod number), PL (pod length), PM (pod mass), SPP (seeds per pod), TSM (total seed mass), and 100-SM (100 seed mass).

**Table 3:** Accumulation of cadmium in roots and shoots of *C. olitorius.*

Harvest Stage (Days after Sowing)	Cd Conc. in the Soil (mg/kg)	Cd Conc. in Plant Parts (mg/kg)	
		Roots	Shoots
44	0	0.0 ^i^	0.0 ^i^
	5	512.5 ^h^	812.5 ^f,g^
	10	920.0 ^d,e,f^	1067.5 ^d^
	15	792.5 ^f,g^	1337.5 ^c^
	20	122.5 ^i^	1425.0 ^c^
85	0	0.0 ^i^	0.0 ^i^
	5	392.5 ^h^	900.0 ^e,f^
	10	685.0 ^g^	1450.0 ^c^
	15	1047.5 ^d,e^	1625.0 ^b^
	20	1342.5 ^c^	1942.5 ^a^
Mean		582	1056.0
CV%		11.8	3.8
LSD		99.5	58.57
*p*-value		<0.001	<0.001

Different letters (a–i) indicate significant differences between cadmium concentrations, different harvest stages, as well as roots and shoots (*p* < 0.05). Cd conc. (Cadmium concentration).

**Table 4:** Translocation of cadmium from roots to aerial parts of *C. olitorius.*

Harvest Stage (Days after Sowing)	Cd Concentration (mg/kg)	Translocation Factor
44	0	0.0 ^e^
	5	1.59 ^c,d^
	10	1.16 ^d^
	15	1.69 ^b,c,d^
	20	11.66 ^a^
85	0	0.0 ^e^
	5	2.30 ^b^
	10	2.12 ^b,c^
	15	1.55 ^c,d^
	20	1.48 ^c,d^
Mean		2.35
CV%		11.9
LSD		0.41
*p*-value		<0.001

Different letters (a,b,c,d and e) indicate significant differences between cadmium concentrations and different harvest stages (*p* < 0.05).

**Table 5:** Properties of the soil used in the research.

Soil Property	Value (mg/kg)
P	10.1
K	169.9
Mg	1432.3
Na	525.4
Zn	22.4
Cu	2.7
Mn	6.0
Fe	395.0
pH	4.5
Clay content	23.0%
Organic matter	4.3%
